# Modifying Faba Bean Protein Concentrate Using Dry Heat to Increase Water Holding Capacity

**DOI:** 10.3390/foods9081077

**Published:** 2020-08-07

**Authors:** Jan M. Bühler, Birgit L. Dekkers, Marieke E. Bruins, Atze Jan van der Goot

**Affiliations:** 1 Wageningen UR, Food & Biobased Research, Wageningen, 6700HB Gelderland, The Netherlands; jan.buhler@wur.nl (J.M.B.); marieke.bruins@wur.nl (M.E.B.); 2 Department of Food Process Engineering, Wageningen University, Wageningen, 6700HB Gelderland, The Netherlands; birgit.dekkers@wur.nl

**Keywords:** food structuring, faba bean, protein functionality, toasting, ingredient functionalization

## Abstract

We investigated the effect of dry-heat treatment on the properties of faba bean protein concentrate using soy protein concentrate as a benchmark. While soy protein—widely used as an ingredient in meat replacers—is recovered through a wet fractionation, protein recovery from starch bearing pulses like faba bean can be done via dry fractionation. This process does not require drying or heating steps and therefore, keeps the original protein functionality intact. This results in differences in properties such as water binding capacity of the protein fraction. Faba bean protein concentrate was dry-heated at temperatures from 75–175 °C, which resulted in higher water-holding capacity and less soluble protein, approaching values of soy protein concentrate. These changes were due to partial denaturation of protein, changing the structure of the protein, and exposing hydrophobic sites. This led to protein aggregation, as observed by light microscopy. Only noncovalent bonds caused the decrease of solubility of dry-heated faba bean protein concentrate. We conclude that dry-heating of dry fractionated faba bean protein can change the functional properties of the protein fraction to desired properties for certain applications. The effect is similar to that on soy, but the underlying mechanisms differ.

## 1. Introduction

In recent years, both research and governing organizations have stressed the need for a protein transition [1]. Currently, soy is the most extensively researched and commercially used crop for plant proteins [2,3]. Soy protein products are widely applied in Asian cuisine and in novel products such as meat analogues. However, a successful protein transition comprises diversification of protein sources. Faba bean could be another plant-based protein source next to soy. It can be grown in colder areas such as northern Europe [4], and is an excellent break crop for wheat-heavy growing cycles [5]. Currently, faba beans are mostly applied in feed applications, but show great potential to be applied in food as well [6], as faba bean protein has good nutritional quality [7]. Faba bean protein can be obtained through dry or wet fractionation, resulting in faba bean protein concentrate (60% protein [8]) or isolate. Faba bean protein has been used in pasta production to increase protein content [9,10,11] without negatively influencing product quality when replacing up to 30% of traditional ingredients. Further applications could include baking [12,13] and use as binders and nutritional enhancers in meat products [14]. It is also used in meat analogue products such as Beyond Sausages (Beyond Meat, El Segundo, CA, USA), Pulled Oats (Gold And Green, Helsinki, Finland), or Crab Free Cakes (Good Catch, New York City, NY, USA). In these products, faba bean is an additive to increase protein content or part of a bean blend [15]. Nevertheless, the current industrial applications are still limited, and therefore faba bean is mostly consumed as an unprocessed side dish [6].

To expand the use of faba bean protein in, for example, meat analogues, its functional properties should be similar to those of soy. Soy is mostly used for structuring applications because of its characteristic functional properties, such as high water-holding capacity (WHC) and good gelling behavior, fat absorption, and emulsification capacities [16]. WHC has been established as a crucial property and is one of the properties often used to describe the functionality of ingredients of meat replacers [17,18,19,20,21]. It has been shown that higher WHC leads to better structuring behavior. Therefore, it was chosen as the focus point of this study. During the purification process, soy protein undergoes a dry-heating or drying step. This impacts water-holding capacity and makes soy suitable for meat replacers [17]. Furthermore, extraction and precipitation conditions can also influence structural and functional properties of soy and faba bean protein [22,23].

Dry fractionation, such as air classification, can be used to create fractions from faba bean that are enriched in protein or starch, as opposed to soy, in which protein is concentrated through a wet process. Dry fractionation is less energy intensive since no water is added, and therefore does not need to be removed, making this process more sustainable and economical [8,24]. Therefore, the protein-rich ingredients are not heated during purification, allowing them to maintain their native state and functionality [8]. Dry processed faba bean proteins have a high solubility in water [24], a low water-holding capacity [25], and the doughs prepared from faba bean proteins are sticky [9], making them difficult to handle [26]. Consequently, the heating step is identified as a potential solution to the low applicability of faba bean protein in meat replacers and possibly other structured food products caused by the less suitable properties for structuring after dry fractionation. Previous studies on dry or mild fractionation processes for faba beans and other legumes often focus on other applications such as emulsions and foams, where high solubility, high emulsification capacity, and high foam stability are required [24,27]. A dry-heat treatment has already been applied by Petitot et al. [10] to change the properties of pasta that contained faba bean protein. They report a stronger structure of the faba bean fortified pasta after a dry-heat treatment and possible Maillard reactions. However, to the best of our knowledge, no investigation was dedicated to explore the possibilities of dry-heating as a tool to improve functional properties such as water-binding capacity of faba bean or to unravel the mechanisms that cause this change in functional properties.

In this research, the influence of a dry-heat treatment on the functional properties important for structuring of faba bean protein concentrate are investigated. The results are compared to a soy protein concentrate. Further, the underlying mechanisms causing the change in functional properties are identified and compared to those in soy protein concentrate.

## 2. Materials and Methods

### 2.1. Materials

Faba bean protein concentrate (FPC, VITESSENCE^TM^ Pulse 3600) was obtained from Ingredion (Hamburg, Germany). Soy protein concentrate (SPC, ALPHA^®^ 8 IP) from Solae (St. Louis, MO, USA) was used as a reference. Their protein content was measured with the Dumas method, using a Nitrogen analyzer, FlashEA 1112 series, (Thermo Scientific, Breda, The Netherlands) and a protein conversion factor of 5.71 [28,29]. FPC had a protein content of 0.60 g g^−1^, SPC 0.675 g g^−1^. Further, the producer reports 0.031 g g^−1^ fat, 0.16 g g^−1^ carbohydrates, 0.11 g g^−1^ dietary fibers, and 0.05 g g^−1^ ash for FPC. All chemicals used were bought from Merck (Breda, The Netherlands).

### 2.2. Dry-Heating

Heating was done dry, in a hot air oven (Heratherm, Thermo Scientific, Breda, The Netherlands) at 75 °C, 100 °C, 120 °C, 130 °C, 150 °C, 160 °C, and 175 °C for 60 min. The protein concentrate powders were spread on a tray as an approximately 5–10 mm-thick layer. The tray was placed in the preheated oven and removed after 60 min. The dry-heated protein concentrates were subsequently placed in a desiccator to cool to room temperature and placed in a closed container for storage before further analysis. Dry heating at all temperatures decreased the water content from 0.0725 g g^−1^ to less than 0.01 g g^−1^.

### 2.3. Water-Holding Capacity and Overall Solubility

To determine the WHC and the solubility of protein and nonprotein fractions of the protein concentrates, a method previously reported by Geerts et al. was used [17]. 0.02 g g^−1^ dispersions were made from the protein concentrates. The dispersions were shaken at room temperature overnight before centrifugation at 10,000× *g* for 30 min at 20 °C (Lynx centrifuge, Thermo Scientific, Breda, The Netherlands). Supernatant was discarded and the mass of the wet pellet was recorded. After freeze-drying, the mass of the dry pellet was also recorded. The solubility of the material was determined according to Equation (1), *WHC_overall_* was determined according to Equation (2), and *WHC_insoluble_* was determined according to Equation (3).

(1) $$solubility=\frac{m_{dry\;powder}-m_{dry\;pellet}}{m_{dry\;powder}},$$

(2) $$WHC_{overall}=\frac{m_{wet\;pellet}-m_{dry\;pellet}}{m_{dry\;powder}},$$

(3) $$WHC_{insoluble}=\frac{m_{wet\;pellet}-m_{dry\;pellet}}{m_{dry\;pellet}},$$

in which *m_dry powder_* is the mass of the overall added dry powder, *m_wet pellet_* is the mass of the pellet after centrifugation and before drying, and *m_dry pellet_* is the mass of the pellet after centrifugation and drying. Since the full material will eventually be used in a final application, the WHC is defined per overall powder instead of per protein. The WHC defined per insoluble material is used to identify the effect of solubility on WHC. Furthermore, protein content of the pellets was measured by the Dumas method, using a Nitrogen analyzer, FlashEA 1112 series (Thermo Scientific, Breda, The Netherlands) and a protein conversion factor of 5.71 to determine protein solubility.

### 2.4. Light Microscopy

An upright microscope Axioscope (Carl Zeiss Microscopy, LLC, Oberkochen, Germany) with a camera was used to inspect the samples on particle scale. Samples were prepared as a 0.02 g g^−1^ dispersion in milliQ water and shaken overnight at room temperature. Subsequently, one droplet of the sample was placed on a microscope glass slide, which was covered with a cover slip. The differences between the non-dry-heated and dry-heated samples were examined by determination of the size of the protein aggregates. Images were taken with 10× and 40× magnification.

### 2.5. SDS-PAGE

A reducing SDS-PAGE (Sodium Dodecyl Sulfate Polyacrylamide Gel Electrophoresis) was performed on all samples. Dispersions of 4 mg L^−1^ of powder were made in water, shaken overnight, then analyzed using SDS-PAGE. 2-Mercaptoethanol was used as a reducing agent in the SDS-PAGE sample buffer. Ready-made BioRad running buffer as well as BioRad precast tris/glycine gels were used. Coomassie BioSafe stain was used to stain the protein bands.

### 2.6. Differential Scanning Calorimetry

Differential Scanning Calorimetry (DSC) was used to determine the state of protein denaturation. Dispersions with 0.15 g g^−1^ dry matter in milliQ water were created and analyzed immediately. About 0.05 g of sample was weighed into High Volume Pans (100 μL, TA Instruments, New Castle, DE, USA) while the weight was accurately recorded and placed in the DSC (DSC-250, TA Instruments). The pans with samples were equilibrated at 20 °C until the temperature was constant, then heated with a ramp of 5 °C/min^−1^ to 125 °C. After cooling, the cycle was repeated once more per sample to verify irreversible denaturation of the protein. Onset temperature and peak height were determined using TRIOS software (TA Instruments). Enthalpy is expressed per total sample mass.

### 2.7. Analysis of Protein–Protein Interaction

Protein–protein interactions were analyzed using a method previously described in [30], with certain changes. A 0.2-mol L^−1^ sodium phosphate buffer (SPB) was used to disrupt electrostatic interactions. The same buffer with the addition of 17.3 mmol L^−1^ sodium dodecyl sulfate (SDS) and 8 mol L^−1^ urea were used to disrupt electrostatic interactions, hydrophobic interactions, and hydrogen bonds. Further, disulfide bonds were disrupted by adding 10-mmol L^−1^ dithiotreitol (DTT) to the second buffer. A total 10 mL of buffer and 0.1 g of protein was mixed and vortexed for 30 s and placed in a rotator for one hour. Afterwards, the solutions were centrifuged for 30 min at 10,000× *g* at 20 °C. The absorption of the supernatant was measured at 280 nm in a Beckman DU720 UV-VIS spectrophotometer (Woerden, The Netherlands). A calibration curve was made by a series of dilutions of Bovine serum albumin dissolved in the specific buffers in the appropriate range. All three buffers were adjusted to pH 6.9 before they were used on the same day they were prepared. Significant difference of the measured values was determined by 1-way ANOVA followed by a Tukey Test with *p* < 0.01.

### 2.8. Hydrophobicity of Insoluble Protein

To measure the hydrophobicity of the insoluble protein, an adaption of a method described by Chelh et al. [31] was used. Sample solutions of 0.02 g g^−1^ were shaken in a rotator for six hours and stored overnight in a fridge. The solutions were centrifuged at 10,000× *g* for 30 min. The supernatant was discarded and pellets were dried for 30 min by placing the tubes upside down on tissue paper. The amount of insoluble protein in the pellets was calculated by measuring and subtracting soluble protein content. This was used to ensure a constant concentration of insoluble protein in the assay. A total of 200 μL of a 1 mg mL^−1^ bromophenol blue (BPB) solution was added to 1 mL of protein dispersion containing 5 mg mL^−1^ of insoluble protein and mixed by a vortex before it was shaken for 15 min in an Eppendorf shaker at 600 rpm. The pH was adjusted to 6.9 for all samples, including the blank. After mixing, the solutions were centrifuged in an Eppendorf 5424 centrifuge (Eppendorf, Nijmegen) for 30 min at 12,000× *g*. The supernatant and a control of milliQ water with 200-μL BPB solution was diluted at a ratio of 1:10 and measured in a Beckman DU720 UV-VIS spectrophotometer (Woerden, The Netherlands) at 595 nm against a blank of milliQ water. The amount of BPB bound was calculated according to Equation (4).

(4) $$BPB_{bound}\;\left(\mu g\;{mg}^{-1}\right)=\frac{200\;\mu g\;*\;(Absorbance_{control}\;-\;Absorbance_{sample})}{Absorbance_{control}\;*\;5\;mg}.$$

### 2.9. Determination of Reducing Sugars

To determine the amount of reducing sugars, a PAHBAH-assay was performed, as described by Lever [32]. To create the PAHBAH-reagent, 0.1 g of 4-Hydroxybenzhydrazide was added to 2 mL of a 0.5 mol L^−1^ HCl solution. Before use, 8 mL of a 0.5 mol L^−1^ NaOH solution was added. A glucose standard curve in the range 150–750 μg mL^−1^ was used. 10% solutions of protein concentrate were made and treated following the same procedure as for determination of WHC, of which the supernatant was used. 200 μL of PAHBAH-reagent was added to 10 μL of supernatant in a 96-Well Elisa Microplate in different dilutions. The microplate was heated for 35 min at 70 °C on a shaker. Absorbance was measured at 405 nm using a Tecan reader 511 (Tecan Benelux).

### 2.10. Determination of Free Amino Groups

The amount of free amino acids present in the soluble protein was measured by OPA (o-Phthaldialdehyde) assay [33]. To obtain OPA-reagent, 3.81 g of borax and 100 mg SDS were dissolved in 80 mL of water, and 80 mg of OPA dissolved in 2 mL of ethanol was added. After dissolving the OPA, 88 mg of DTT was added and the solution was filled up to 100 mL. The solution was filtered using a 0.45-μm filter. A L-serine calibration curve of 50–200 mg L^−1^ was used. Samples were made and treated following the same procedure as for determination of WHC, of which the supernatant was used. 1.5 mL of OPA-reagent was mixed with 200 μL of supernatant. The samples were measured after 3 min at 340 nm using a Beckman DU720 UV-VIS spectrophotometer (Woerden, The Netherlands). The number of free amino groups per raw material was adjusted for protein solubility.

### 2.11. Statistical Analysis

Statistical analysis was performed using R in RStudio. The number of repetitions (n) is reported with the results. Significant differences of the measured values were determined by 1-way ANOVA followed by a Tukey Test with *p* < 0.05 unless stated otherwise. All differences discussed are significant, unless stated otherwise. Furthermore, significant differences are indicated by different small letters in the figures.

## 3. Results & Discussion

### 3.1. Influence of Dry-Heating on Solubility and Water Holding

**Figure 1:**
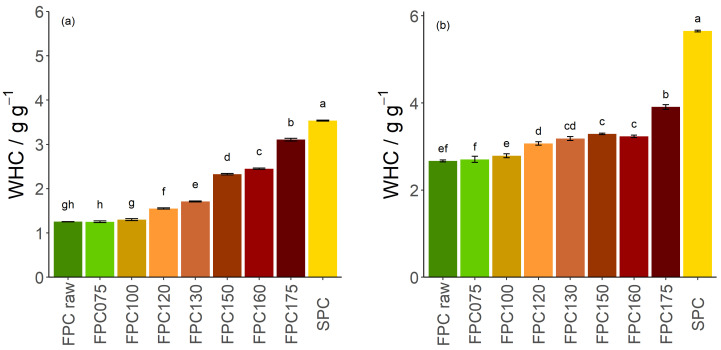
(**a**) Water-holding capacity of the overall powder (Equation (2)) and (**b**) water holding capacity of the insoluble fraction (Equation (3)) of dry-heated and non-dry-heated faba bean protein concentrate (FPC) as well as commercial soy protein concentrate (SPC). *n* = 3. Water-holding capacity (WHC) of FPC powder dry-heated at up to 100 °C did not show any significant difference from non-dry-heated FPC powder. FPC powder dry-heated at higher temperatures showed an increase in WHC, with 150 °C and 175 °C having the largest effect. The WHC of the insoluble fraction only increased slightly after heat treatment, with the exception of 175 °C (**b**).

The overall WHC of non-dry-heated FPC was 1.25 g g^−1^, less than half of the value of SPC (3.53 g g^−1^, Figure 1a). While dry-heating at 75 °C and 100 °C did not alter the overall WHC, dry heating at higher temperatures led to a significant increase in WHC. Substantial increases are seen for samples treated at 150 °C and 175 °C, reaching values of 3.10 g g^−1^.

**Figure 2:**
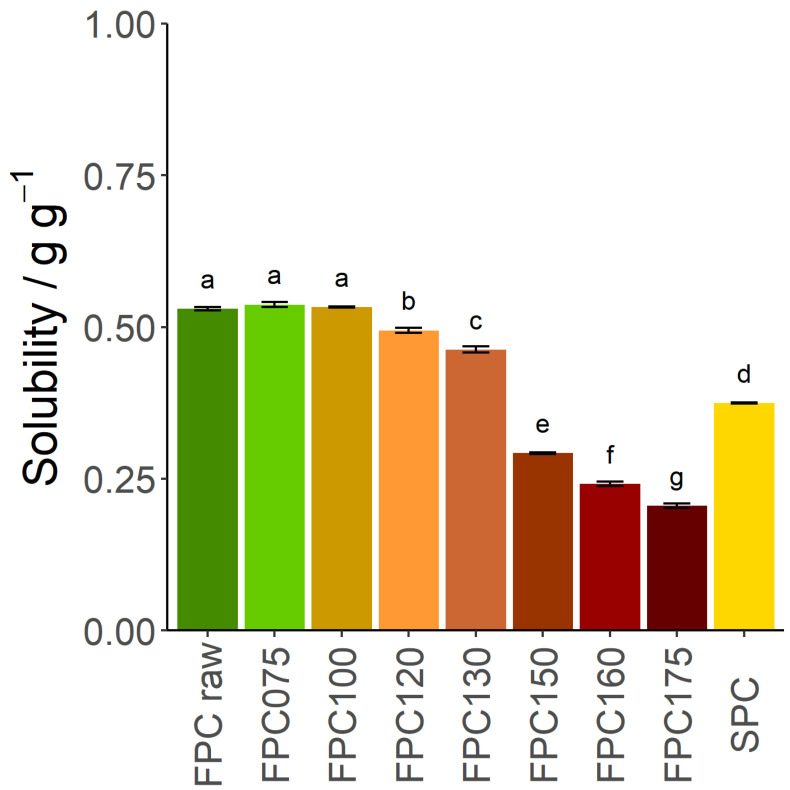
Solubility of the material of dry-heated and non-dry-heated FPC and commercial SPC. *n* = 3. Dry-heating at 75 °C and 100 °C had no influence on the solubility. Dry-heating at 120 °C and 130 °C slightly lowered solubility; while dry-heating at 150 °C, 160 °C, and 175 °C cut solubility in half. SPC is shown as an industry reference.

To understand this change in WHC, the solubility of FPC was investigated. The amount of soluble material was initially 0.53 g g^−1^ for the non-dry-heated sample, remaining unchanged after dry-heating at 75 °C and 100 °C, and only slightly decreasing after dry-heating at 120 °C and 130 °C. However, after dry-heating at 150 °C or more, it decreased to 0.29 g g^−1^, lower than the solubility of SPC (Figure 2). The WHC of the insoluble fraction only increased by 23.2% after dry-heating at 150 °C (Figure 1b), whereas the WHC of the overall dry matter increased by 86.4%. As the soluble protein fraction has no WHC, the increase of the insoluble protein fraction is the main cause of the increase in WHC of FPC dry-heated at 150 °C. At 175 °C, the change in overall WHC cannot be explained by insolubility only.

An explanation for the decrease in solubility of heat-treated FPC could be a change of the protein structure due to degradation leading to aggregation of proteins. Considering the composition of the FPC, sugars or starches could have had an influence on the solubility of the protein fraction, e.g., through an intermolecular reaction, such as Maillard reactions. To study the underlying mechanisms, three samples were selected: non-dry-heated, mildly dry-heated (100 °C), and severely dry-heated (150 °C) FPC. Raw FPC and SPC were also further analyzed as blank and industry reference.

### 3.2. Light Microscopy

**Figure 3:**
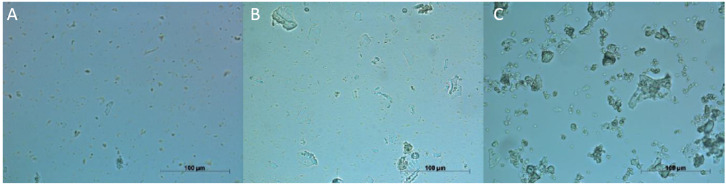
Light microscopy pictures of 0.02 g g^−1^ dispersions of dry-heated and non-dry-heated FPC. Samples dry-heated at 150 °C (**C**) showed larger particles than non-dry-heated FPC (**A**) and FPC dry-heated at 100 °C (**B**).

To visualize protein aggregation, dry-heated and non-dry-heated samples were observed using light microscopy. Figure 3 shows a 40× magnification of the non-dry-heated (A) and dry-heated samples at 100 °C (B) and 150 °C (C). For the non-dry-heated sample, black spots below 10 μm were observed, with no particles above 20 μm. After dry-heating at 100 °C and 150 °C, the number of larger aggregates increased, with sizes above 20 μm. Dry-heating at 100 °C resulted in fewer particles in the sample, with multiple particles below 10 μm. Samples dry-heated at 150 °C contained more particles above 20 μm and 50 μm, while the small particles that were observed in the first two samples were hardly found. Therefore, it is an indication that dry-heating at 100 °C and 150 °C led to aggregation of particles, and the effect was stronger at the higher temperature.

### 3.3. Molecular Weight of Protein Subunits

**Figure 4:**
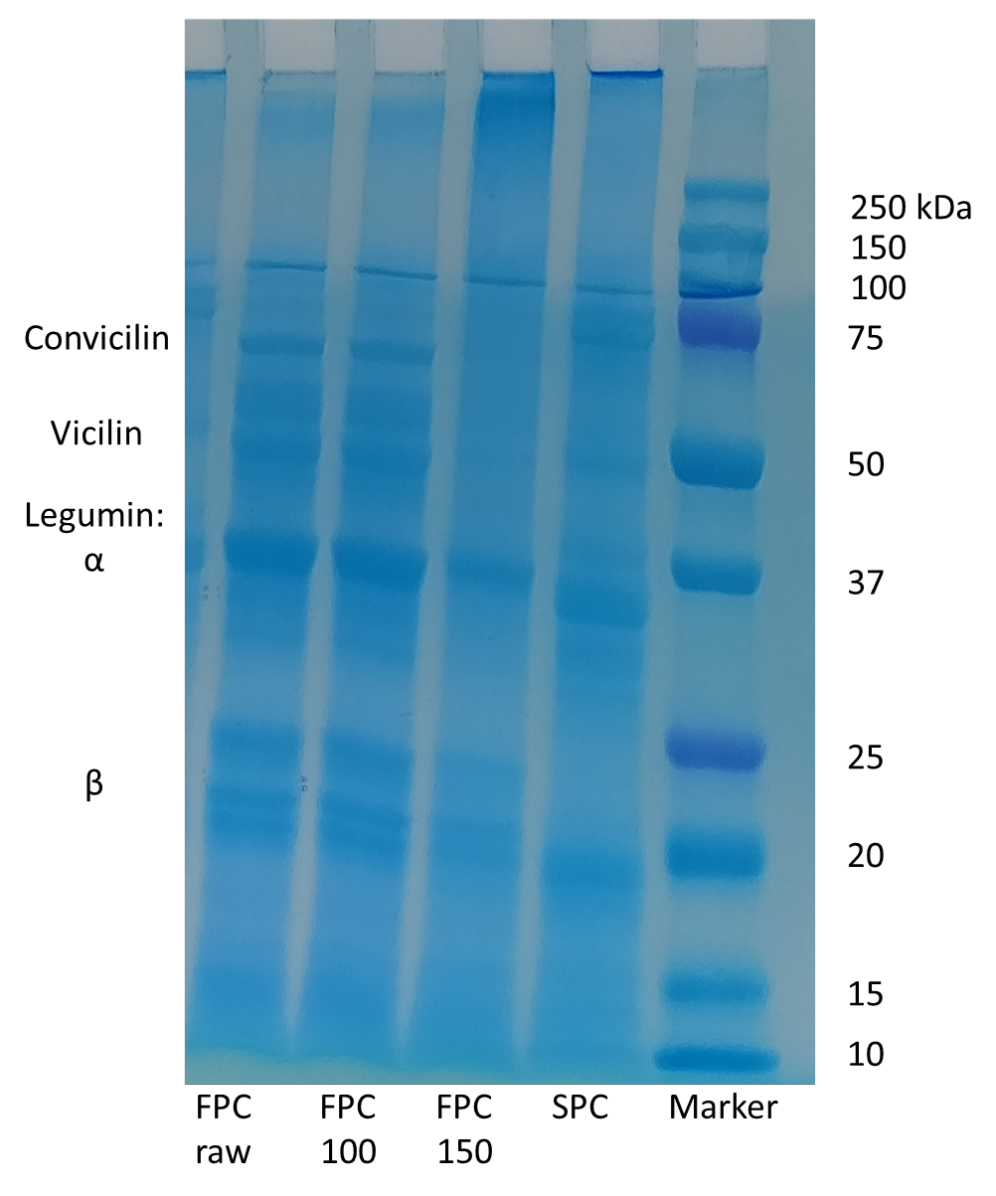
SDS-PAGE of non-dry-heated and dry-heated FPC. SPC is shown as a reference. Non-dry-heated and dry-heated at 100 °C FPC showed bands for convicilin, vicilin, α-legumin, and β-legumin. FPC dry-heated at 150 °C showed the legumin bands less pronounced, while the vicilin and convicilin bands were not detectable. Instead, indistinguishable bands larger than 250 kDa appeared. For soy, the subunits for glycinin and β-conglycinin were visible.

To determine the change in molecular weight of the protein subunits of legumin and vicilin in FPC, SDS-PAGE was performed (Figure 4). For non-dry-heated FPC as well as FPC dry-heated at 100 °C, bands corresponding to convicilin (60 kDa), vicilin (46–55 kDa), α-legumin (38–40 kDa), and β-legumin (23 kDa) were seen [34]. The bands for α- and β-legumin were less intense after dry-heating at 150 °C, while the bands for vicilin and convicilin disappeared. Instead, indistinguishable bands larger than 250 kDa appeared at the top of the column. SPC showed bands corresponding to the subunits of glycinin and β-conglycinin [35]. SDS-PAGE showed aggregation of proteins. It also showed that no peptide bonds were broken due to dry-heating, as the subunits of the FPC proteins stayed intact and no bands appeared at the bottom of the column.

### 3.4. Nonenzymatic Browning Reactions

**Figure 5:**
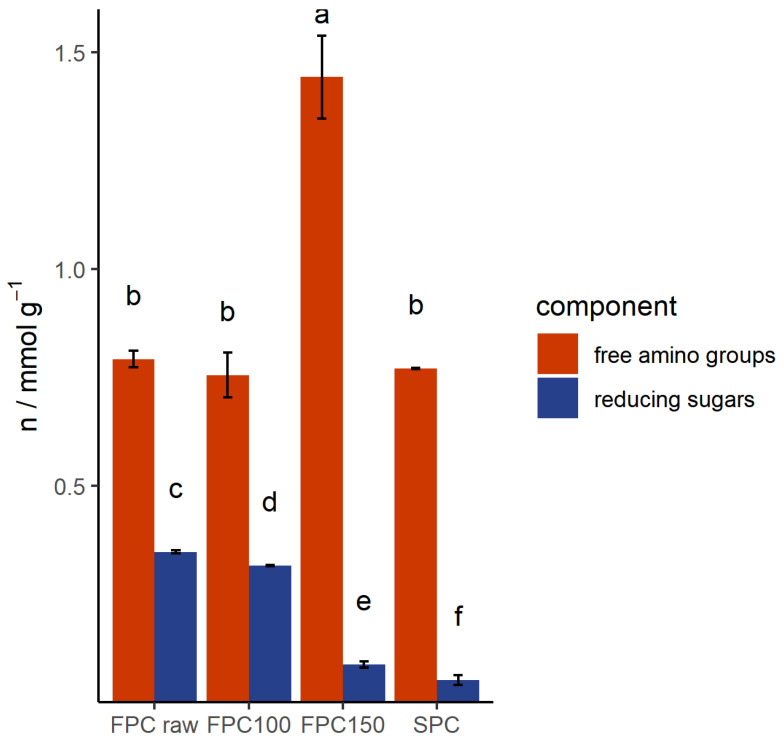
Number of reducing sugars and free amino groups in mmol g^−1^ dry matter of dry-heated and non-dry-heated FPC and commercial SPC. *n* = 3. The number of reducing sugars decreased with increasing dry-heating temperature, while the number of free amino groups increased after dry-heating at 150 °C. SPC is shown as an industry reference.

During heat treatments, coloration reaction such as caramelization of sugars or Maillard reactions can occur. As mentioned in Section 3.1, Maillard reactions could affect the solubility of proteins [36,37,38]. In Figure 5, the number of reducing sugars per total dry matter is shown. FPC dry-heated at 100 °C had 8% less reducing sugars than non-dry-heated FPC. Dry-heating at 150 °C caused a more pronounced reduction of reducing sugars by 0.26 mmol g^−1^. This illustrates that caramelization occurred.

The number of free amino groups measured in the non-dry-heated FPC (0.79 mmol g^−1^) was in line with the generally expected amount of free amino groups containing amino acids in FPC (lysin and arginine, 0.62 mmol g^−1^) [39]. The loss of free amino groups can be used as a measure of Maillard reactions [36,40,41]. As reducing sugars react with free amino groups during Maillard reactions, a similar decrease in both the free amino groups and reducing sugars was expected if Maillard reactions occurred. However, the number of free amino groups did not decrease, but increased by 0.65 mmol g^−1^ after dry-heating at 150 °C (Figure 5). The increase of free amino groups might have been caused by a change in protein conformation, which could have caused more exposure of the free amino groups. Protein hydrolyzation is unlikely, according to the results of the SDS-PAGE.

Thus, no direct evidence of Maillard reactions was found. However, as Maillard reactions occur mostly in wet conditions [42], the decrease of reducing sugars is probably attributed to caramelization. Since caramelization only involves sugars and not proteins, its effect on protein aggregation is negligible. Further, the decrease in reducing sugars limits Maillard reactions in any final processing steps that involve heating.

### 3.5. Protein–Protein Interactions

**Figure 6:**
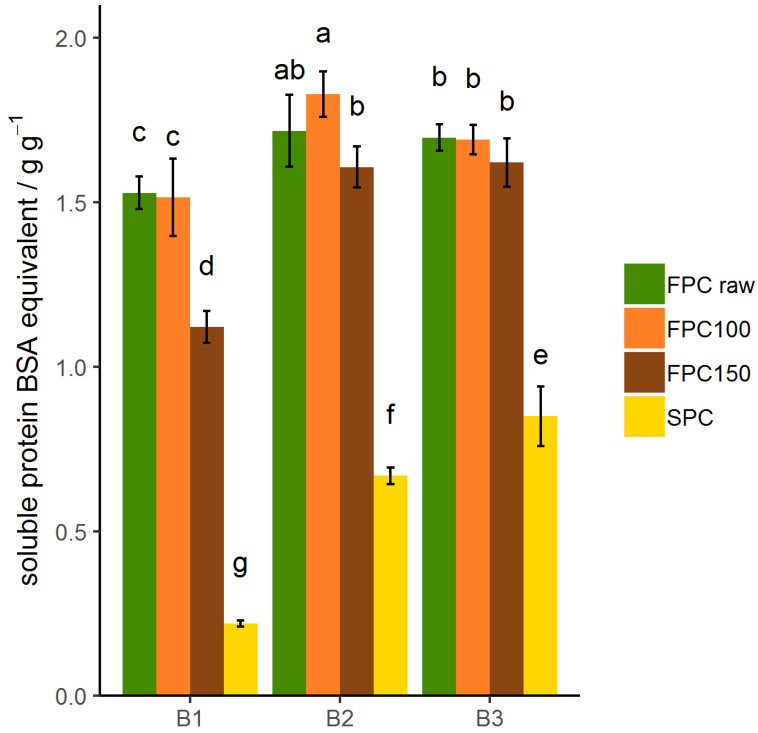
Solubility of protein dry-heated and non-dry-heated FPC as well as commercial SPC after disrupting electrostatic interactions (B1), noncovalent interactions (hydrophobic and electrostatic interactions and hydrogen bonds) (B2), and covalent and noncovalent interactions (B3). *n* = 9. Solubility is expressed as BSA equivalent per gram FPC or SPC. Significant differences are indicated by different lowercase characters (*p* < 0.01). Samples dry-heated at 150 °C showed a significant difference in solubility in B1 compared to non-dry-heated FPC. Solubility of dry-heated and non-dry-heated FPC increased from B1 to B2, but not from B2 to B3. Solubility of SPC increased from B1 over B2 to B3.

Different selective agents were used to solubilize the protein to find the type of bonds stabilizing the protein and causing lower solubility after dry-heating. In sodium phosphate buffer (B1), electrostatic interactions were disrupted by the phosphate buffer, which led to a solubility of the non-dry-heated FPC of 1.53 g g^−1^ (Figure 6). In sodium phosphate buffer + SDS + urea (B2), noncovalent bonds such as H-bonds and hydrophobic interactions were also disrupted. This increased the solubility of non-dry-heated FPC significantly to 1.71 g g^−1^, illustrating the presence of noncovalent bonds. By addition of DTT (B3), disulfide bonds were also broken. The solubility of non-dry-heated FPC in B1 was not significantly different than in B2. The solubility of FPC dry-heated at 100 °C showed no significant difference to the non-dry-heated FPC in any of the buffers. Solubility of FPC dry-heated at 150 °C was lower than that of non-dry-heated FPC in B1, but not in B2 or B3. Since no increase was detected from B2 to B3 for any material, disulfide bonds do not contribute to the insolubility of FPC. The similar trend for solubility in water showed that in FPC, only noncovalent bonds were affected by dry-heating at 150 °C and contributed to the insolubility of these samples. This is in line with the findings of Zheng et al. [43], who showed that purified legumin from faba bean formed aggregates after a heat treatment that were stabilized by noncovalent bonds.

Solubility of SPC increased from B1 to B2 and to B3 (Figure 6), showing that both noncovalent and covalent bonds keep it from solubilizing. Similar trends have been reported for SPI [44], SPC [30], and wheat gluten [45,46]. Chen et al. [47] state that the importance of noncovalent bonds outweighs that of covalent bonds after extrusion of SPI. This is in line with the findings of Liu and Hsieh [48], who found that disulfide bonds did not play an important role for solubility of SPI and SPC as such, but caused insolubility after extrusion.

The amino acid composition of SPC [49] and faba bean [39] are comparable. In fact, the largest difference lies with Arginine, of which faba bean has 25% more than SPC. All other amino acids are within 0.01 g g^−1^ of overall protein. However, it is possible that they have different bonds that stabilize the protein, as they are organized in different structures: vicilin and legumin in faba bean; and glycinin and β-conglycinin in soy. Vicilin lacks the disulfide bond forming the amino acid cystein [50,51,52] compared to β-conglycinin [53,54], whereas legumin from faba bean is very similar to glycinin [55,56,57]. This explains the limited influence of disulfide bonds on the solubility of faba bean protein concentrate. Further, the lack of disulfide bonds presents an explanation for the difference in solubility and also overall WHC between raw FPC and SPC. The inherent inability of faba bean proteins to form disulfide bonds is also a potential reason why dry-heat treatment only increased the WHC of FPC, but did not close the gap to SPC.

**Figure 7:**
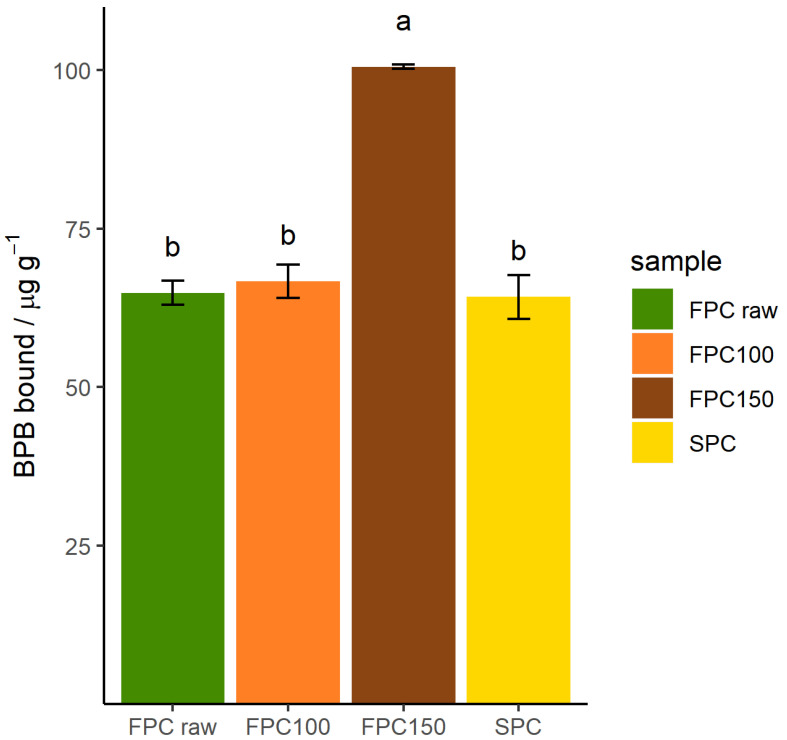
Hydrophobicity as determined by amount of bromophenol blue (BPB) bound to insoluble particles of dry-heated and non-dry-heated FPC as well as commercial SPC. *n* = 3. FPC dry-heated at 150 °C bound more BPB than all other samples.

Additionally, hydrophobicity of insoluble particles was determined by the amount of BPB bound. As can be seen in Figure 7, dry-heating FPC at 100 °C did not affect the amount of BPB bound and therefore hydrophobicity. SPC bound the same amount of BPB as non-dry-heated FPC, coincidentally. However, FPC dry-heated at 150 °C bound 55% more BPB than non-dry-heated FPC, showing that the treatment increased the hydrophobicity of the insoluble fraction. This suggests that the hydrophobic sites on the inside of the protein were exposed by (partial) denaturation.

### 3.6. Denaturation of Protein

**Figure 8:**
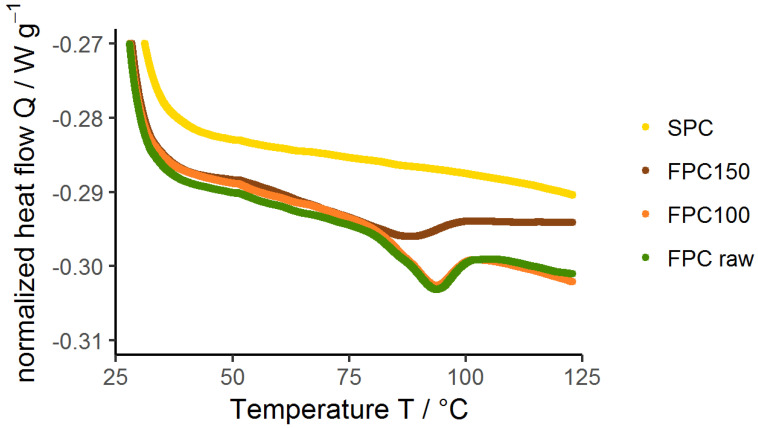
Thermograms of 0.15 g g^−1^ dispersions of dry-heated and non-dry-heated FPC as well as commercial SPC, produced with Differential Scanning Calorimetry (DSC). A ramp of 5 °C was used and each sample was measured in two cycles. The second cycles did not show any peaks for any sample.

DSC measurements were conducted to determine the extent of the protein denaturation due to dry-heating. Figure 8 is an example of a DSC thermogram of the performed measurements for every treatment and ingredient. For all samples, an endothermic peak was observed in the first run. The second run showed no peaks for all samples. An overview of the curve analysis of the experiments performed in triplicate can be found in Table 1. The *T_d_* found for non-dry-heated FPC at a dry matter content of 0.15 g g^−1^ was 93.2 °C, with a Δ*H* of 0.92 J g^−1^. The enthalpy Δ*H* as well as the peak temperature *T_d_* decreased for samples dry-heated at 150 °C. Since less heat was needed to denature these samples, they must have been partially denatured by the dry-heating treatment. Similar findings have been reported by Artfield et al. [58], who also found a decrease in Δ*H* necessary for denaturation of faba bean concentrate after wet-heat treatments. They concluded that partial and complete, irreversible denaturation occurred due to treatment at 85 °C and 95 °C, respectively. This is in line with the *T_d_* found by them, 88 °C, and in this research, 93.2 °C. Shevkani et al. [59] found *T_d_* ranged from 82.7 °C to 85.5 °C for different varieties of faba bean protein isolates, Sosulski et al. [60] found *T_d_* = 91 °C for faba bean protein flour, all at comparable dry matter contents. The differences in *T_d_* can be explained by differences in amino acid composition of the used faba bean varieties or the state of protein due to processing history resulting in a different protein structure [61,62]. It is hypothesized that the partial denaturation caused by dry-heating at 150 °C changed the structure of the proteins so that more hydrophobic sites were exposed, causing the proteins to aggregate and become less soluble in water. This hypothesis is supported by the results of the analysis of protein–protein interactions using different buffer systems and the hydrophobicity study on insoluble proteins.

## 4. Conclusions

Dry-heating can be a useful tool trying to bridge the gap between the functional properties of mild fractionated plant proteins such as faba bean protein concentrate and conventionally processed SPC. In this study, it was shown that it had a similar positive effect on WHC of FPC as it did for SPC, Dry heating is a tool to control the functional properties of FPC. Increased heating leads to higher WHC and lower solubility. These changes make the FPC a potential ingredient to replace soy in applications like meat replacers. However, soy still has a higher WHC. While the aggregation/insolubility of FPC after dry-heating was caused by hydrophobic interactions and hydrogen bonds, SPC was further stabilized by disulfide bridges. The absence of these disulfide bridges in FPC explains its higher solubility, and therefore, potentially, the remaining difference in WHC with soy. Finally, this study shows that when choosing ingredients for meat replacers, not only protein content and source but also (thermal) processing history should be taken into consideration.

## Figures and Tables

**Table 1:** Average peak temperature and enthalpy of protein denaturation of dry-heated and non-dry-heated FPC. *n* = 3. Values obtained using the TA Instruments software TRIOS. Dry-heating at 150 °C shifted the peak to a lower temperature and reduced the enthalpy to a third of the value of non-dry-heated FPC.

	*T_d_*/°C	+/−/°C	Δ*H*/J g^−1^	+/−J g^−1^
FPC raw	93.2	0.2	0.92	0.05
FPC100	93.6	0.5	0.96	0.03
FPC150	88.8	0.6	0.32	0.08

## Abbreviations

The following abbreviations are used in this manuscript:

BPB	Bromophenol Blue
DSC	Differential Scanning Calorimetry
DTT	Dithiotreitol
FPC	faba bean Protein Concentrate
HCL	Hydrochloride
m	mass
NaOH	Sodium Hydroxide
OPA	o-Phthaldialdehyde
PAHBAH	4-Hydroxybenzhydrazide
SDS	Sodium Dodecyle Sulfate
SDS-PAGE	Sodium Dodecyl Sulfate Polyacrylamide Gel Electrophoresis
SPB	Sodium Phosphate Buffer
SPC	Soy Protein Concentrate
WHC	Water-Holding Capacity
